# The physiological basis for genetic variation in water use efficiency and carbon isotope composition in *Arabidopsis thaliana*

**DOI:** 10.1007/s11120-013-9891-5

**Published:** 2013-07-28

**Authors:** Hsien Ming Easlon, Krishna S. Nemali, James H. Richards, David T. Hanson, Thomas E. Juenger, John K. McKay

**Affiliations:** 1 Department of Land, Air & Water Resources, University of California, Davis, CA 95616 USA; 2 Department of Biology, University of New Mexico, Albuquerque, NM 87131 USA; 3 Section of Integrative Biology, University of Texas, Austin, TX 78712 USA; 4 Department of Bioagricultural Sciences & Pest Management, Colorado State University, Fort Collins, CO 80523 USA; 5 Present Address: Department of Plant Sciences, University of California, Davis, CA 95616 USA; 6 Present Address: Monsanto Company, Jerseyville, IL 62052 USA

**Keywords:** ABI4, Carbon isotope composition, Mesophyll conductance, Photosynthetic capacity, Stomatal conductance

## Abstract

Ecologists and physiologists have documented extensive variation in water use efficiency (WUE) in *Arabidopsis thaliana*, as well as association of WUE with climatic variation. Here, we demonstrate correlations of whole-plant transpiration efficiency and carbon isotope composition (δ^13^C) among life history classes of *A. thaliana.* We also use a whole-plant cuvette to examine patterns of co-variation in component traits of WUE and δ^13^C. We find that stomatal conductance (*g*_s_) explains more variation in WUE than does *A*. Overall, there was a strong genetic correlation between *A* and *g*_s_, consistent with selection acting on the ratio of these traits. At a more detailed level, genetic variation in *A* was due to underlying variation in both maximal rate of carboxylation (*V*_c_max) and maximum electron transport rate (*J*max). We also found strong effects of leaf anatomy, where lines with lower WUE had higher leaf water content (LWC) and specific leaf area (SLA), suggesting a role for mesophyll conductance (*g*_m_) in variation of WUE. We hypothesize that this is due to an effect through *g*_m_, and test this hypothesis using the abi4 mutant. We show that mutants of ABI4 have higher SLA, LWC, and *g*_m_ than wild-type, consistent with variation in leaf anatomy causing variation in *g*_m_ and δ^13^C. These functional data also add further support to the central, integrative role of ABI4 in simultaneously altering ABA sensitivity, sugar signaling, and CO_2_ assimilation. Together our results highlight the need for a more holistic approach in functional studies, both for more accurate annotation of gene function and to understand co-limitations to plant growth and productivity.

## Introduction

The efficiency with which plants fix CO_2_ relative to their rate of H_2_O loss is called water use efficiency (WUE), and when high, WUE can mitigate the tradeoff between CO_2_ uptake and H_2_O loss. In C_3_ plants, low stomatal conductance (*g*_s_) minimizes water loss (transpiration, *E*) and can be a rapid and effective strategy; however, it results in reduced CO_2_ uptake (*A*) and growth (Schulze 1986; Geber and Dawson 1997; Condon et al. 2002). Genetically based variation in WUE has been documented in both crops and non-cultivated species (McKay et al. 2003; Hall et al. 2005). Physiologists are interested in intrinsic WUE (*A*/*g*_s_) as a tool for studying how the fundamental trade-off of losing water for gaining CO_2_ is regulated by stomatal and other physiological adjustments (Buckley and Mott 2002; Comstock 2002). Evolutionary biologists have studied variation in WUE as it is likely an important component of local adaptation (Donovan and Ehleringer 1994; Heschel et al. 2002; Geber and Griffen 2003; Caruso et al. 2005). Likewise, plant breeders have long considered WUE an important target (Passioura 1977).

WUE can be estimated in a variety of ways at various spatio-temporal scales, including with lysimeter studies, gas exchange measurements, or stable carbon isotope composition. Tissue carbon isotope composition is an increasingly popular approach, and its advantages include integration over long periods of gas exchange and development, amenability to high throughput sampling, relatively low cost, and high heritability. Stable carbon isotope composition of leaves (δ^13^C) (the ratio of the amount of ^13^C to ^12^C isotopes in a sample relative to a standard), provides a time-integrated estimate of intrinsic WUE (Farquhar et al. 1989; Dawson et al. 2002). In *Arabidopsis thaliana* (here after *Arabidopsis*), common garden experiments have identified substantial variation in δ^13^C among natural accessions and some of this variation likely represents local adaptation to climate (McKay et al. 2003, 2008; Juenger et al. 2005, 2010; Christman et al. 2008; Monda et al. 2011; Des Marais et al. 2012; Lasky et al. 2012). In addition, QTL have been identified for δ^13^C (Juenger et al. 2005; Masle et al. 2005; McKay et al. 2008).

In plant breeding, WUE is an important target of selection, although the complexity of the trait, and difficulty of phenotyping has prevented many breeding programs from attempting to select on WUE directly (Araus et al. 2002). Many studies have shown variation in δ^13^C among cultivars. In crops, one particularly successful example is an Australian wheat breeding program, where selection on δ^13^C in a greenhouse environment led to new varieties that had increased yield in semiarid rainfed conditions (Rebetzke et al. 2002). Conversely, in conditions where water is not limiting, selection for reduced WUE may lead to greater yields (Passioura 1977; Fischer et al. 1998).

Although it is heritable, appears to be under selection in nature, and may correlate with yield in C_3_ crops (Condon et al. 1987), the mechanistic basis of genetic variation in δ^13^C is still unclear. Variation in δ^13^C can be due to variation in photosynthetic biochemistry, conductance of CO_2_ to the leaf interior and chloroplast, or a combination of these (Seibt et al. 2008). Thus, similar leaf δ^13^C and similar WUE can evolve via mutations that cause low *A* with low conductance or mutations that cause high *A* with proportionally higher conductance (Farquhar et al. 1989). This is further complicated because conductance from ambient air to the interior of the leaf is influenced both by *g*_s_ and additional variability of conductance into leaf mesophyll cells and chloroplasts (*g*_m_), which can change over the long-term with leaf morphology (von Caemmerer and Evans 1991; Evans et al. 1994, 2009; Tosens et al. 2012) and over the short-term through changes in protein-mediated chloroplast membrane permeability (Flexas et al. 2006; Uehlein et al. 2008; Heckwolf et al. 2011). When examining the combined effects of *g*_s_ and *g*_m_, it is important to recognize that they operate in series rather than in parallel and that the regulation of *g*_m_ is poorly understood. Within a genotype, *g*_s_ and *g*_m_ usually respond in a correlated way to environmental stimuli (Flexas et al. 2007, 2008; Warren 2008; Barbour et al. 2010) although, opposite responses have also been observed (Galle et al. 2012). Patterns of genetic covariation of *g*_s_ and *g*_m_ have not been investigated. However, it is known that variation in *g*_m_ contributes to leaf carbon isotope discrimination, further increasing the importance of considering *g*_s_ and *g*_m_ in interpretations of δ^13^C (Warren and Adams 2006; Barbour et al. 2010).

Understanding the physiological basis of variation in δ^13^C and intrinsic WUE is important for improving plant productivity and understanding the evolution of wild species. Here, we report a series of experiments designed to investigate a mechanistic understanding of the physiological basis of variation in intrinsic WUE in *Arabidopsis*. At the coarse level, we can ask if variation in intrinsic WUE is primarily due to variation in *A* or *g*_s_. For example, threefold variation in *g*_s_ and twofold variation in leaf *N* concentration among natural accessions of *Arabidopsis* suggest substantial variation in *g*_s_ and *A* may separately or in concert be responsible for the observed variation in δ^13^C (Christman et al. 2008; Des Marais et al. 2012). Des Marais et al. (2012) found large differences in physiology between life history classes in *Arabidopsis*. Although, the Des Marais study focused on variation in gene expression, they also reported constitutive variation in leaf structural traits between life history classes. Winter annual types had higher intrinsic WUE. This is consistent with coordinated selection on WUE, *A*, and *g*_s_ and life history observed in other species (Geber and Dawson 1997). Higher WUE was associated with lower leaf water content (LWC) and specific leaf area (SLA) (Des Marais et al. 2012). Taken together, these results suggest that increased leaf density is associated with higher photosynthetic capacity (Terashima et al. 2011), but may come at the cost of lower stomatal and mesophyll conductance to CO_2_ (Parkhurst and Mott 1990; Evans et al. 1994; Syvertsen et al. 1995; Kogami et al. 2001).

Studies in *Arabidopsis* have identified extensive natural variation in plant–water relations and gas exchange physiology (Juenger et al. 2005, 2010; Masle et al. 2005; Bouchabke et al. 2008; Christman et al. 2008; McKay et al. 2008; Monda et al. 2011; Des Marais et al. 2012; Pons 2012). The present study was undertaken to examine natural variation in leaf physiological traits that are the likely cause of the observed variation in δ^13^C and associated WUE parameters in natural accessions of *Arabidopsis*, and to determine if these traits vary independently or co-vary in a coordinated and predictable manner. First, we tested if the expected relationship between transpiration efficiency (shoot dry mass/transpiration; TE) and leaf δ^13^C was present in 96 natural accessions of *Arabidopsis*. In a smaller set of 18 natural accessions spanning the range of variation in δ^13^C, we measured rosette *A*, *g*_s_, and intercellular CO_2_ concentration (*C*_i_) and examined the relationship of *C*_i_ and δ^13^C. To further characterize natural variation in *A*, we examined maximal carboxylation rate (*V*_c_max) and photosynthetic electron transport rate (*J*max) in three accessions using photosynthetic carbon dioxide response curves (Sharkey et al. 2007). Additionally, we used gas exchange measurements coupled with online isotopic measurements to determine instantaneous carbon isotope discrimination using tunable diode laser spectroscopy (TDL) (Flexas et al. 2006; Barbour et al. 2007; Heckwolf et al. 2011) to estimate *g*_m_ in stomatal regulation mutants to investigate the relationship of these mechanistically related traits (Warren et al. 2003; Yamori et al. 2006).

## Materials and methods

### δ^13^C and transpiration efficiency (Experiment 1)

Our first goal was to use a relatively high throughput approach to look for variation and co-variation across the species range. 96 natural accessions were selected from the native range of *Arabidopsis* to evaluate plant biomass production and water use (Nordborg et al. 2005). Individual plants were grown in 250-mL plastic cups, each filled with a standard mass of 1:1 fritted clay and Promix BT potting soil mix. We measured field capacity of the soil mix following a 24-h gravitational drain of saturated soil. Each cup was covered with parafilm and sealed with a plastic lid that had a 6-mm diameter hole. Two replicates of each of 96 ecotypes were planted and cold stratified in the dark for 7 days at 4 °C. Plants were grown in two independent growth chambers at 200 μmol m^−2^ s^−1^ PPFD in a randomized block design. Photoperiod was 12 h light/12 h dark and the temperature cycled 23/18 °C (light/dark). Every 2 days, each container was weighed and additional water was added with a syringe to bring the soil in each container to 90 % field capacity. Total transpiration (*E*_total_) was summed for the 35 days growing period for each experimental plant. Plants were harvested, and aboveground material was oven dried and weighed (DW). We assessed evaporative loss from the containers using “blanks” lacking an *Arabidopsis* plant. Total evaporation from the blank containers was <4 % of the average *E*_total_ from pots in the experiment. Transpiration efficiency (TE) of each plant was calculated as DW/*E*_total_. Dried leaves were ground to a fine powder and δ^13^C was determined at the UC Davis Stable Isotope Facility (http://stableisotopefacility.ucdavis.edu/). When grown outside in free air, the use of carbon isotope discrimination, Δ, is preferred (Farquhar et al. 1982), but when growth chamber and greenhouse studies are included the value of air δ^13^C is uncertain and variable, thus requiring the use of leaf δ^13^C instead of Δ. Differences in δ^13^C within the same experiment indicate differences in intercellular CO_2_ concentration, but δ^13^C must be viewed with caution when comparing different experimental conditions.

### Whole-shoot gas exchange (Experiment 2)

To follow up on the patterns from the 96 accessions, 18 natural accessions of *Arabidopsis* were used in whole-shoot gas exchange experiments to evaluate the physiological basis of variation in δ^13^C. Eleven of the accessions were spring annuals, and seven were winter annuals. Four replicates of each genotype were grown in a growth chamber in a randomized block design. Each plant was grown in a pot constructed from a 50-mL centrifuge tube with the bottom cut off and “planted” in a 164-mL Conetainer™ pots (Stuewe and Sons, Corvallis, OR) filled with a 1:1 mixture of potting mix (Sunshine mix, Sun Gro Horticulture, Bellevue, WA) and fritted clay. After planting, pots were cold stratified at 4 °C for 7 days, then transferred to a growth chamber. Photoperiod was 12 h with 350 μmol m^−2^ s^−1^ PPFD and temperature was cycled 23/20 °C (light/dark).

**Fig. 1:**
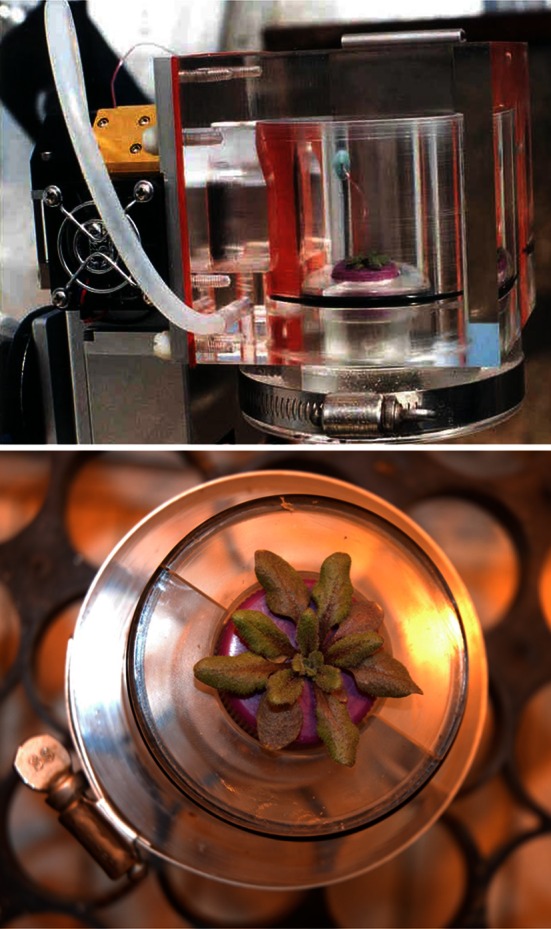
Cuvette used for whole-plant gas exchange measurements. The cuvette is mounted on the LI-6400 IRGA and cuvette control system (*gold*-*plated panel*, *fan* and *aluminum box*, *upper photograph*). This system allows accurate, rapid measurement of CO_2_ (*A*) and H_2_O (*E*) exchange of whole shoots of *Arabidopsis* plants. The whole-plant cuvette incorporates a leaf temperature thermocouple that interfaces directly with the LI-6400. Intrinsic WUE (*A/g*_s_), stomatal conductance (*g*_s_), internal CO_2_ concentration (*C*_i_), and other variables can be calculated from these measurements. All interior surfaces are Teflon coated or Ni-plated, the cuvette has extremely low leak rates when operated in lab conditions with high external CO_2_, and the circular design provides excellent mixing using the LI-6400 fans. Plants can be rapidly changed using multiple inserts (*lower photo*)

Instantaneous whole-canopy gas exchange rate was measured using a LI-6400 (Li-Cor Inc., Lincoln, NE, USA) with a custom-made whole-shoot *Arabidopsis* cuvette (Fig. 1). Cuvette PPFD was maintained at 350 μmol m^−2^ s^−1^ PPFD, CO_2_ was maintained at 400 μmol mol^−1^, and temperature and relative humidity were set to growth chamber conditions. Each block was measured on a different day, 28–31 days after sowing. Following measurements for each plant, leaf area was determined from digital photographs of the rosette using Scion Image (Scion Corporation, Frederick, MD, USA).

*A*:*C*_i_ responses were measured for three accessions (Tsu-1, SQ-8, and Kas-1) which differed in *A* and δ^13^C. Cuvette conditions were the same as above, but light was increased to 1,000 μmol m^−2^ s^−1^ PPFD. Photosynthetic carbon dioxide response curves were measured on four rosettes of each accession. The number of replications of *A*:*C*_i_ measurements were limited by chamber environment equilibration time at each CO_2_ set point. The least squares iterative curve-fitting procedure (Sharkey et al. 2007) model was used to fit Farquhar et al.’s (1980) biochemical model of photosynthesis and obtain maximal carboxylation rate (*V*_c_max) and maximal photosynthetic electron transport rate (*J*max).

### Leaf water content (Experiment 3)

39 natural accessions from the native range of *Arabidopsis* previously used in Mckay et al. (2003) were measured for LWC and leaf δ^13^C. Four replicates of each ecotype were grown in a greenhouse at UC Davis in a randomized block design. Seeds were sown in 250-mL pots in peat-based potting mix with slow-release fertilizer and vernalized at 4 °C for 5 days. Day length was extended to 16 h using supplemental lighting at 350 μmol m^−2^ s^−1^ PPFD. Greenhouse mean relative humidity and air temperature were 44 % and 23 °C, respectively. Shoots were harvested at the initiation of flowering and shoot fresh weight (FW) was determined, leaf area was determined from scans of dissected rosettes using Scion Image (Scion Corporation, Frederick, MD, USA), and shoots were dried and weighed (DW). Entire dried shoots were ground and processed for carbon isotope analysis at the UC Davis Stable Isotope Facility (http://stableisotopefacility.ucdavis.edu/). LWC (%) was calculated as 100 × (FW − DW)/DW.

### Mesophyll conductance (Experiment 4)

*Arabidopsis* seeds of ecotype Columbia and the abi4 mutant provided by the Arabidopsis Biological Resource Center (Columbus, OH, USA) were used for leaf mesophyll conductance to CO_2_ (*g*_m_) experiments. Seven replicates of each genotype were grown in a growth chamber in a randomized block design. Photoperiod was 12 h with 350 μmol m^−2^ s^−1^ PPFD and temperature was cycled 23/20 °C (light/dark). A LI-6400 (Li-Cor Inc., Lincoln, NE, USA) with whole-shoot *Arabidopsis* cuvette (Fig. 1) was coupled with online isotopic measurements of CO_2_ entering and leaving the shoot chamber to determine instantaneous carbon isotope discrimination and *g*_m_ using TDL (Flexas et al. 2006; Barbour et al. 2007; Heckwolf et al. 2011). Calculations for *g*_m_ were based on whole-shoot gas exchange measurements at 350, 700, and 175 (μmol m^−2^ s^−1^) PPFD using the slope-based approach given in Evans et al. (1986). Shoots were harvested after gas exchange, leaf area was determined from rosette photographs using Scion Image (Scion Corporation, Frederick, MD, USA), and shoots were dried and weighed (DW). LWC (%) was calculated as above and SLA was calculated as rosette area/DW.

### Statistical analysis

We analyzed phenotypic data for physiological traits using standard fixed effect ANOVAs with the Proc GLM in SAS (SAS Institute 1999). We estimated correlations among physiological traits as the standard Pearson product-moment correlation between genotype means.

In the case of the TE experiment, we analyzed phenotypic data for physiological traits using a linear mixed model analysis with the Proc Mixed procedure in SAS (SAS Institute 1999). We fit a model including accessions as a random effect and chamber, experiment, and their interaction as fixed effects. The variance component for the random effect was estimated using restricted maximum likelihood (REML) and assessments of significance were based on likelihood ratio tests (Little et al. 1996). We obtained empirical best linear unbiased predictors (BLUPs) associated with the random effects and consider these breeding values for each accessions. BLUPs are robust estimates of the impact of a particular accession on the measured trait while controlling for the fixed effects (chamber and experimental run). For TE, we fit a model that included both chamber and experimental run as a fixed effect. For δ^13^C, we fit a simpler model including accession as a random variable and experimental run as a fixed effect. In this case, factors associated with chamber could not be included because replicates within each experimental run were pooled for mass spectroscopy analysis. All subsequent analyses involving TE and δ^13^C rely on BLUP estimates. The TE and δ^13^C values were normally distributed and residuals from analyses did not exhibit heteroscedasticity.

We estimated broad-sense heritability by computing the ratio *V*_G_/*V*_P_, where *V*_G_ equals the among-accession variance component and *V*_P_ equals the total phenotypic variance for the study phenotypes. We estimated genetic correlations (*r*_G_) among TE and δ^13^C as the standard Pearson product-moment correlation between genotype means or BLUPs.

## Results and discussion

### Variation in TE and δ^13^C

**Fig. 2:**
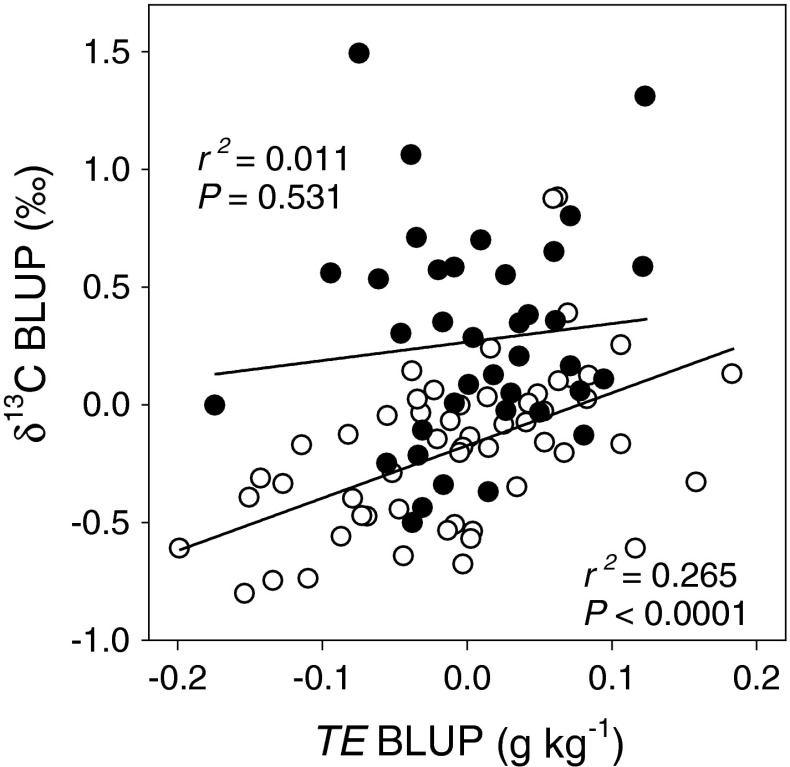
Relationships of transpiration efficiency (TE) and leaf carbon isotope composition (δ^13^C) among 96 natural accessions of *Arabidopsis thaliana.* *Symbols* represent best linear unbiased predictors (BLUPs) associated with breeding values for each accession (see text). *Open* and *filled* *symbols* represent spring and winter accession means, respectively. *Lines* represent linear regression; *r* ^2^ and *P* values are given

The 96 natural accessions of *Arabidopsis* in experiment 1 (Table 1) exhibited considerable variation in time-integrated measures of water use efficiency, i.e., whole-plant TE and δ^13^C. We observed a 3.33 g kg^−1^ and 5.12 ‰ range of variation in TE and δ^13^C among accessions, respectively, (TE mean = 2.02 ± 0.28 g kg^−1^) (δ^13^C mean = −30.64 ± 0.90 ‰). In both cases, we observed significant broad-sense heritability (TE, *H* ^2^ = 0.09, accession *P* = 0.031; δ^13^C, *H* ^2^ = 0.667, accession *P* = 0.001). For the experiment 1, we found replication block, growth chamber, and their interaction were significant sources of environmental variation in TE (in all cases, *P* < 0.005). Likewise, we found that the replication block was a significant source of environmental variation for δ^13^C (*P* < 0.0001). Despite the low heritability of the TE data, our experimental design and analysis allowed us to estimate breeding values as BLUPs. Spring accessions fit the expected positive relationship between TE and δ^13^C (*r*_G_^2^ = 0.265, *P* < 0.0001, Fig. 2). The winter annuals had greater intrinsic WUE as indicated by δ^13^C than the spring annuals, but this was not related to TE (*r*_G_^2^ = 0.011, *P* = 0.531, Fig. 2). Together these data suggest that variation in δ^13^C is likely due to stomatal limitations (on *C*_i_) in the spring accessions, but in winter accessions, other mechanisms (like *g*_m_) not affecting water loss may be leading to variation in δ^13^C (Seibt et al. 2008). Alternatively, variation in root carbon allocation unaccounted for in TE may explain the observed pattern in winter accessions. In principle, the greater belowground allocation in winter accessions could result in lower TE without affecting δ^13^C, but this hypothesis remains to be tested.

**Table 1:** Summary of experiments

Experiment	Genotypes	Measurements	Conditions
Experiment 1	96 natural accessions representing a range of latitudes, elevations and climates.	TE, δ^13^C	200 μmol m^−2^ s^−1^ PPFD, 12 h photoperiod
Experiment 2	Ag-0, Bil-5, Bur-0, C24 Col-2, Eden-1, Got-22, HR5, Kas-1, Knox-18, Ler-1, NFA-10, Omo2-3, Sq-8, Tamm-2, Ts-1, Tsu-1, Ws-2	Whole shoot gas exchange (*A*, *g*_s_, *C*_i_), δ^13^C, *V*_c_max, *J*max	350 μmol m^−2^ s^−1^ PPFD, 12 h photoperiod
Experiment 3	Aa-0, Ag-0, Cvi-0, Kas-1, Mh-0, Ms-0, Di-g, Est, Ws-3, Kondara, Da(1)-12, Hodja-Obi-garm, Je54, Petergof, Rubezhnoe-1, Sn(5)-1, Sorbo, An-1, Bch-3, Can-0, Db-1, Edi-0, Ei-4, En-1, Et-0, Jl-3, Ka-0, Mrk-0, Pi-0, Rd-0, Rsch-4, Sei-0, Ta-0, Wl-0, Wei-1, Tsu-1, Rld-2, Oy-1, Shahdara	LWC, δ^13^C	350 μmol m^−2^ s^−1^ PPFD, 16 h photoperiod
Experiment 4	abi4-1 (At2g40220), Columbia	Whole shoot gas exchange with online carbon isotope discrimination (*A*, *g*_s_, *C*_i_, *g*_m_, SLA, LWC)	350 μmol m^−2^ s^−1^ PPFD, 12 h photoperiod

### Variation in components of WUE

**Fig. 3:**
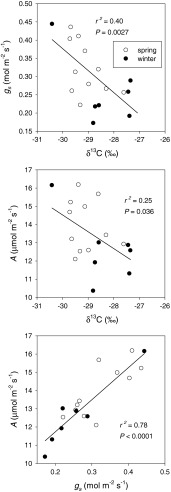
Relationships between assimilation (*A*), stomatal conductance (*g*_s_), and leaf carbon isotope composition (δ^13^C) at 350 μmol photons m^−2^ s^−1^ from whole-shoot gas exchange of 18 accessions of *Arabidopsis* selected from the larger panel of accessions to represent extremes in δ^13^C. *Open* and *filled symbols* represent spring and winter accession means, respectively. *Lines* represent linear regression; *r* ^2^ and *P* values are given

The 18 natural accessions of *Arabidopsis* in experiment 2 were selected to represent a wide range of intrinsic WUE as indicated by δ^13^C (Table 1). Whole-plant gas exchange measurements in a custom cuvette (Fig. 1) showed that these lines also exhibit considerable variation in whole rosette *A* and *g*_s_ in a common environment (Fig. 3). Accession mean whole rosette *A* ranged between 10 and 16 μmol m^−2^ s^−1^, but the heritability was not significantly different from zero (*P* = 0.137). *g*_s_ showed significant genetic variation, ranging between 0.17 and 0.45 mol m^−2^ s^−1^ with a heritability of *H* ^2^ = 0.33 (accession *P* value = 0.002). In addition, *g*_s_ was a better predictor of variation in δ^13^C than *A*. We found a significant negative correlation between δ^13^C and *g*_s_ among accessions (*r* ^2^ = 0.40, *P* = 0.0027), and a weaker correlation between δ^13^C and *A* (*r* ^2^ = 0.25, *P* = 0.036). In general, the high conductance lines had low intrinsic WUE, as indicated by δ^13^C, but there was a wide range of δ^13^C in the low conductance lines, suggesting additional sources of variation. The expected negative correlation between δ^13^C and *g*_s_ was largely caused by the spring accessions. The winter accessions tended to show the opposite pattern (not significant), with the exception of Tamm-2, an accession from Finland that had the highest *g*_s_ of all.

Despite the lack of heritability of *A* and the weak correlation of *A* with δ^13^C, we did find a significant positive correlation between *g*_s_ and *A* among accessions (*r* ^2^ = 0.78, *P* = 0.00001). This is consistent with the optimization of stomatal regulation to maximize carbon gain while minimizing the water loss (Katul et al. 2010). Accessions that have high conductance should be under selection for increased biochemical capacity (Bloom et al. 1985). Although, it is not formally stated, such optimality approaches interpret consistent patterns of correlation in physiological traits (Reich et al. 1997) as evidence of selection optimizing their ratios or covariances (Donovan et al. 2011). Under such a scenario, selection would favor mutations that lead to a co-limitation of *g*_s_ and RuBP utilization and regeneration.

**Fig. 4:**
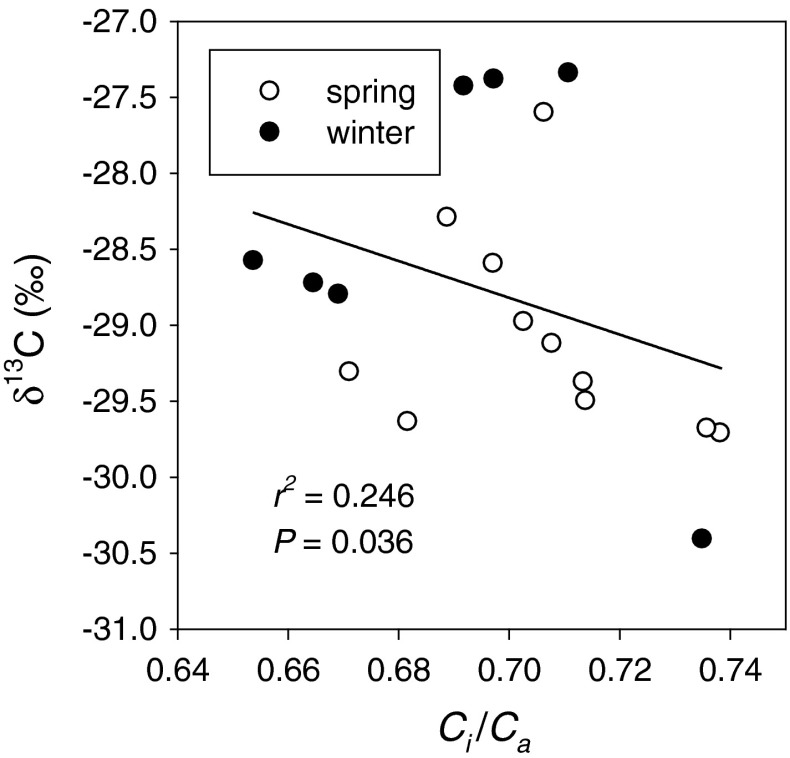
Relationship between the ratio of intercellular to atmospheric partial pressure CO_2_ (*C*_i_/*C*_a_) at 350 μmol photons m^−2^ s^−1^ and carbon isotope composition (δ^13^C). *Open* and *filled symbols* represent spring and winter accession means, respectively. *Line* represents linear regression; *r* ^2^ and *P* values are given

In general, winter *Arabidopsis* accessions had lower *g*_s_ and *A* than spring *Arabidopsis* accessions. Across accessions there was large variation in *C*_i_ */C*_a_, but it was only weakly related to δ^13^C (Fig. 4). No consistent difference in *C*_i_ */C*_a_ was seen between the winter and spring annuals.

**Fig. 5:**
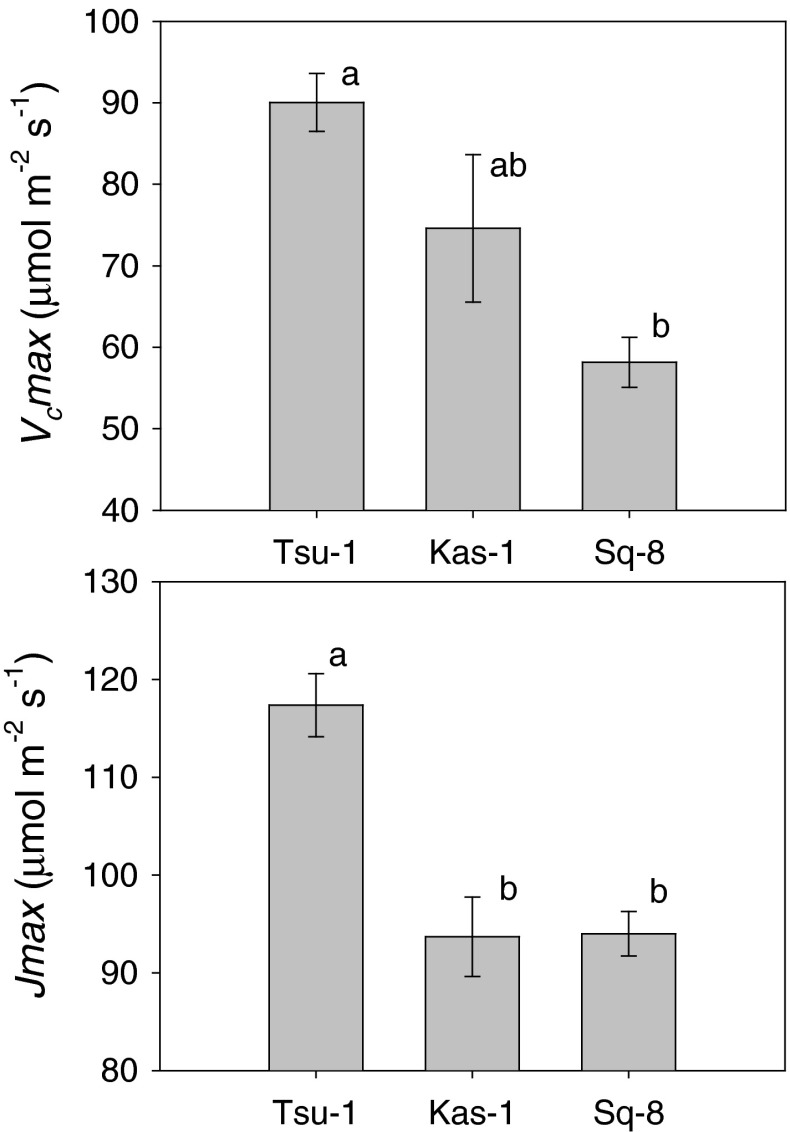
Maximum carboxylation rate of rubisco (*V*_c_max) and maximal photosynthetic electron transport (*J*max) obtained from photosynthetic carbon dioxide response curves in three accessions (Tsu-1, Sq-8, and Kas-1) which differed in *A*. Each *bar* represents the mean ± SE (*n* = 4) for each accession. *Letters* represent significant differences among accessions. Genotype F-ratio = 12.14 and *P* = 0.0078 for *V*_c_max. Genotype F-ratio = 11.01 and *P* = 0.0098 for *J*max

The overall finding of experiment 2 was that accessions with low *g*_s_ and high δ^13^C had lower *A* compared to low δ^13^C accessions. Overall, these data are consistent with large effects of *g*_s_ on δ^13^C, but the weaker correlation of *C*_i_ and δ^13^C suggest a more complex mechanism than predicted by theory. To better understand processes limiting photosynthesis in *Arabidopsis* accessions, we conducted detailed CO_2_ response curves of assimilation for low and high WUE spring accessions Tsu-1 and SQ-8 and high WUE winter accession Kas-1. Maximum carboxylation rate of rubisco (*V*_c_max) was higher in low WUE Tsu-1 (δ^13^C = −29.7) than Sq-8 (δ^13^C = −28.6) (*P* = 0.01), as expected (Fig. 5). Similar, maximal photosynthetic electron transport (*J*max) was also higher in Tsu-1 than Sq-8 or Kas-1 (δ^13^C = −28.8) (*P* = 0.002, *P* = 0.002).

The major biochemical limitations to photosynthesis, *V*_c_max and *J*max, appeared optimized to accessions’ *C*_i_ as indicated by δ^13^C. *V*_c_max and *J*max were lower in low *g*_s_, high WUE accessions operating at lower *C*_i_. The higher ratio of *V*_c_max to *J*max in Kas-1 compared to Sq-8 suggests a lack of limitation by *J*max under the low *g*_s_ typical of Kas-1. Simultaneous changes in *V*_c_max and *J*max are consistent with a limitation of photosynthesis by RuBP utilization and regeneration (Farquhar and Sharkey 1982). Likewise, proportional changes in components of photosynthetic apparatus and *g*_s_ suggest acclimation of these processes are closely coupled (Cowan 1986).

### Variation in structure

**Fig. 6:**
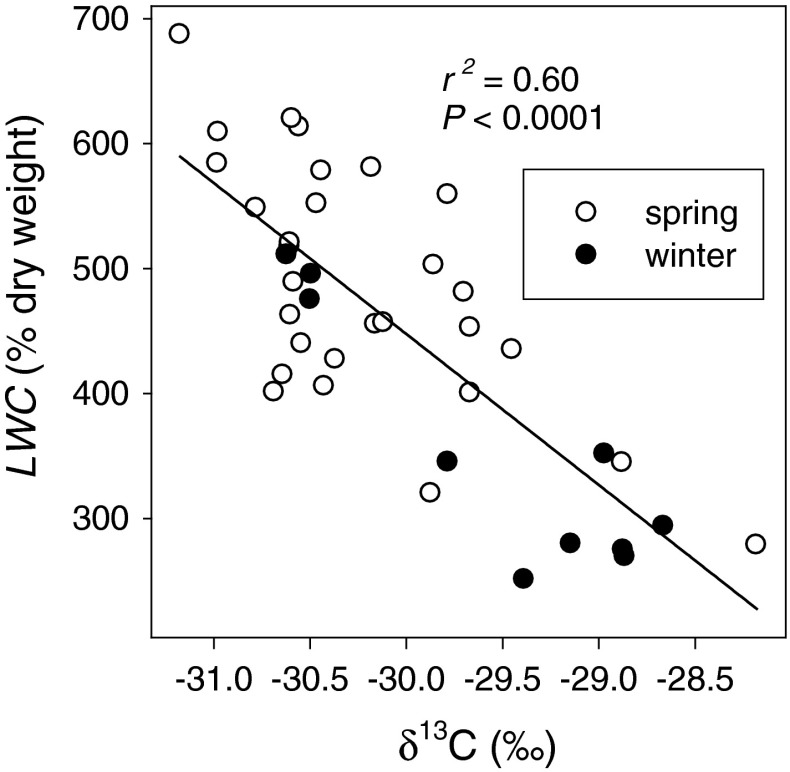
Relationship between leaf water content (LWC) and leaf carbon isotope composition (δ^13^C) among 39 accessions of *Arabidopsis thaliana. Open* and *filled symbols* represent spring and winter accession means, respectively. *Line* represents linear regression; *r* ^2^ and *P* values are given

In experiment 3, we examined 39 natural accessions of *Arabidopsis* for variation in δ^13^C and LWC (Table 1). We found a significant negative correlation between δ^13^C and LWC among accessions (*r* ^2^ = 0.6, *P* < 0.0001). Spring accessions tended to have higher LWC and lower WUE, as indicated by δ^13^C, than winter accessions. Accession differences in LWC most likely result from the effect of mesophyll cell wall thickness on leaf density and not differences in water potential as plants in experiment 3 were not water stressed (Garnier and Laurent 1994; Evans et al. 1994). Leaf anatomical traits such as leaf and cell wall thickness, surface area of mesophyll cells exposed to internal air spaces, and the location of chloroplasts within those cells was initially shown to correlate with *g*_m_ several decades ago (von Caemmerer and Evans 1991; Evans et al. 1994). In particular, mesophyll cell wall thickness was shown to negatively affect *g*_m_. Therefore, high LWC accessions should have thinner mesophyll cell walls resulting in high *g*_m_ and more negative δ^13^C (Evans et al. 1994), which is consistent with our data. These ideas have been revisited recently and the importance of the cell wall properties (thickness and water content) and the coverage of air exposed surfaces of mesophyll cells by chloroplasts is receiving more attention (Evans et al. 2009; Tholen and Zhu 2011; Tosens et al. 2012). Direct measurement of leaf thickness and density may explain some of the variation in *g*_m_ and δ^13^C among plants with similar LWC values (Fig. 6). Alternatively, variation in COO-porin content or activity could be responsible for the *g*_m_ and δ^13^C variation in plants with LWC. Recent studies have found a significant role for chloroplast membrane CO_2_ transporting aquaporins (COO-porin) has been demonstrated and provides a clearly heritable mechanism for both rapid and sustained adjustment of *g*_m_ (Flexas et al. 2006; Uehlein et al. 2008, 2012; Heckwolf et al. 2011). We have found strong correlations between LWC, *A*, and *g*_s_, so focusing on plants with similar LWC should limit the influence of those factors on variation in δ^13^C and increase the relative influence of *g*_m_ from cell wall properties or COO-porin content or activity on δ^13^C variation.

### The ABI4 transcription factor causes changes in leaf anatomy and mesophyll conductance

To further test for a causal effect of leaf anatomy on gas exchange (experiment 4 in Table 1), we used abi4, a mutant of locus AT2G40220, which is an AP2/ERF transcription factor (TF). ABI4 is closely related to the DREB2 TFs and the mutant was initially described as ABA insensitive based on a germination screen (Finkelstein 1994). Subsequent work has shown that the transcript is expressed in seedlings (Soderman et al. 2000) and fully developed rosette leaves (Finkelstein et al. 1998). In addition to its key role in ABA signaling, further characterization of this transcription factor has proposed a large and diverse set of functions including sugar signaling and response (Husijer et al. 2000; Bossi et al. 2009), and root development (Signora et al. 2001; Shkolnik-Inbar and Bar-Zvi 2011). There are hundreds of loci whose expression is altered in the ABI4 mutant (Kerchev et al. 2011). Given that it is a transcription factor, this is not surprising, but does illustrate the challenge of functional annotation of such pleiotropic loci.

**Fig. 7:**
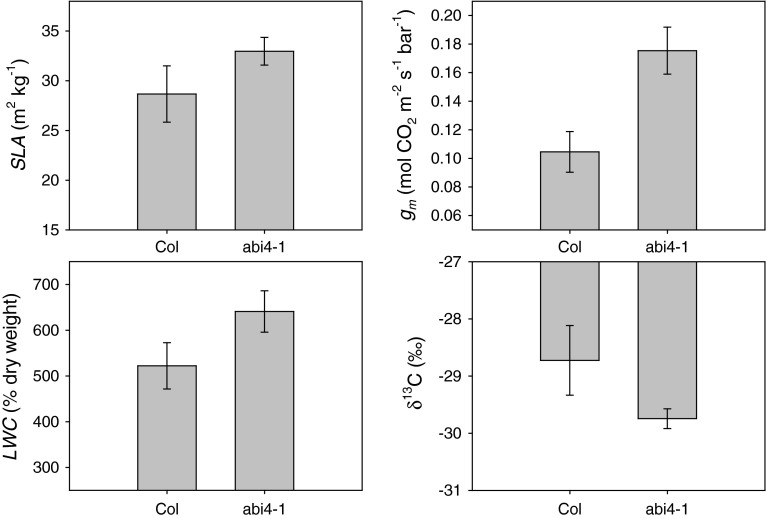
Comparison of specific leaf area (SLA), leaf water content (LWC), mesophyll conductance (*g*_m_), and leaf carbon isotope composition (δ^13^C) between abi4-1 and Columbia (Col) wildtype. Each *bar* represents the mean ± SE (*n* = 7) for each genetic line. *P* < 0.05 for *g*_m_, SLA, LWC, and δ^13^C

abi4 had higher SLA and LWC than wildtype, revealing a novel effect of this TF on leaf anatomy. In addition, abi4 had increased *g*_m_ and more negative δ^13^C, consistent with the idea that SLA causes variation in δ^13^C via effects on *g*_m_ (Fig. 7). The correlation of SLA, *A*, and *g*_s_ with LWC helps to explain why LWC is strongly correlated with leaf gas exchange, i.e., LWC appears to be an inverse proxy for cell wall thickness. When taken together, our data show that *Arabidopsis* leaves trade-off high WUE for low *A*, by trading off leaf anatomy based diffusional CO_2_ limitation with water loss through stomata. Essentially, plants with the highest *A* achieve this via the combination of high *g*_s_ and thin leaves (high SLA). High *g*_s_ keeps *C*_i_ high and the thin leaves have cells with thin walls. Thin walls increase *g*_m_ and keeps CO_2_ concentration at the sites of carboxylation (*C*_c_) high (Evans et al. 1994). Conversely, when photosynthesis is directly limited by the combination of cool winter temperatures and high light through effects on electron transport, then low *g*_s_ would be selected for to improve WUE. We hypothesize that thicker leaves would provide more internal shading and more efficient light use, further decreasing *g*_m_ and *C*_c_ explaining the winter annual phenotype.

Although, a few of the AP2/ERF transcription factors in *Arabidopsis* have been the subject of detailed study, there are 122 of these loci in *Arabidopsis* (Nakano et al. 2006) and much remains unknown about their function. Recent studies have revealed increasingly complex roles for members of this transcription factor family. For example, a recent study identified eight AP2/ERFs induced by photorespiration (Foyer et al. 2012). This, combined with the known roles of ABI4 in sugar signaling to photosynthesis including repression of RBCS (Van Oosten et al. 1997; Teng et al. 2008), and our results showing effects on leaf density and *g*_m_, are expanding this picture.

## Conclusions

Detailed measurements on a diverse set of accessions detail the traits underlying natural variation in intrinsic WUE and carbon isotope composition. Previous studies have shown that spring accessions have lower intrinsic WUE than accessions with winter life histories. Proportional changes in *A*, *g*_s_, *V*_c_max, and *J*max suggest acclimation of these processes are closely coupled. We also show strong covariation between LWC and δ^13^C, where spring annuals tend to have higher LWC and lower intrinsic WUE. We hypothesize that this is due to an effect through *g*_m_, and test this hypothesis using the abi4 mutant. The abi4 mutant shows increased SLA and reduced *g*_m_ compared to the wildtype, consistent with the pattern of covariance found in the natural accessions.

Previous separate studies in *Arabidopsis* have addressed variation in δ^13^C, plant–water relations, leaf anatomy, and photosynthetic capacity and limitations, including *g*_m_. Here, we use a whole canopy approach to examine variation and covariation in all of these components. As predicted by optimality, these traits are not independent, but instead covary as would be expected if selection and photosynthetic acclimation favors states of colimitation. In addition, we show that perturbation of a single transcription factor leads to this trait covariance. This emphasizes the need for whole plant approaches and high dimensional phenotyping to accurately annotate the gene function.
